# Interpreting match performance in elite futsal: considerations for normalizing variables using effective time

**DOI:** 10.3389/fspor.2023.1256424

**Published:** 2023-09-04

**Authors:** Konstantinos Spyrou, João Nuno Ribeiro, António Ferraz, Pedro E. Alcaraz, Tomás T. Freitas, Bruno Travassos

**Affiliations:** ^1^ UCAM Research Center for High Performance Sport, UCAM Universidad Católica de Murcia, Murcia, Spain; ^2^ Facultad de Deporte, UCAM Universidad Católica de Murcia, Murcia, Spain; ^3^ Strength and Conditioning Society, Murcia, Spain; ^4^ Research Center in Sports Science, Health Sciences and Human Development (CIDESD), Department of Sport Sciences, University of Beira Interior, Covilhã, Portugal; ^5^ CIFD, Sports Research and Training Center, Jean Piaget University of Angola, Luanda, Angola; ^6^ NAR Nucleus of High Performance in Sport, São Paulo, Brazil; ^7^ Portugal Football School, Portuguese Football Federation, Lisbon, Portugal

**Keywords:** five-a-side soccer, team-sports, match play, external load, tactical performance

## Abstract

**Introduction:**

This study aimed to investigate the physical and individual technical-tactical performance of elite futsal players.

**Methods:**

Thirteen National Team futsal players (27.4 ± 4.7 years, 70.3 ± 7.6 kg, 176.3 ± 5.5 cm) competed in 15 official matches in three distinct competitions. Session rate of perceived exertion (sRPE) and player load (PL) were measured for assessing physical internal and external load, respectively. Individual tactical actions were analyzed using Instat Scout®, considering variables such as fouls, shots, shots on target, goals, successful dribbles, passes, successful passes, ball losses, ball recoveries, and challenges won.

**Results:**

The cluster analysis classified the players into two groups: “high-involvement” (HIGH) and “low-involvement” (LOW), based on their average effective playing time, sRPE, and PL. A linear mixed model was used to examine the differences in external and internal load and technical-tactical variables between the two groups, accounting for individual repeated measures. Considering absolute values, HIGH players presented higher sRPE, RPE, total PL, shots, goals, passes, successful passes, ball losses and recoveries, and challenges won (*p* = 0.001–0.039; ES = 0.43–2.48), when compared to their LOW counterparts. However, when analyzed relative to effective time, LOW players reached higher total PL and shots per minute (*p* = 0.001–0.012; ES = 0.39–0.93) when compared to HIGH players. Non-significant differences were found for the rest of the variables.

**Discussion:**

In summary, these results emphasize the importance of normalizing internal and external load variables and technical-tactical performance variables per effective playing time in futsal. The current findings indicate that players with lower involvement can present the same or even higher physical and technical-tactical performance compared to teammates with more playing time.

## 1. Introduction

Futsal performance results from the combination of different high-intensity intermittent actions (e.g., accelerations, decelerations, changes of direction) to accomplish specific tactical demands (1, 2). Due to futsal's nature (i.e., unlimited substitutions, space and time restriction, and the constant variations in the contexts of play), previous research was conducted on the influence of interchange rotations and playing time on match demands of elite futsal players (3). Specifically, Ribeiro et al. (3) found that players can maintain high-intensity efforts during the match rotations over the entire time of the match. Notably, while players with more playing time and a work–rest ratio equal to or greater than one exhibited a greater capacity to repeat high-intensity efforts, while, players with less playing time and a work–rest ratio below one tended to decrease the number of such actions.

Despite the previous work analyzed physical demands between players by considering high-intensity actions, to further understand individual player's performance there is a need to also consider individual tactical actions according to the game dynamics (4–6). In fact, there has been a lack of a thorough research analyzing the relationship between physical internal and external load variables and individual tactical actions in relation to game dynamics and time of play (both absolute and relative) (5). A recent systematic review (5) demonstrated that mainly both 1 vs. 1 and 5 vs. 4 with fly-goalkeeper situations have been analyzed in futsal, and only few studies have analyzed the collective tactical behavior during match play. This knowledge gap restricts our understanding of how these factors influence both player and team performance. Therefore, further research is warranted to investigate the difference between absolute and relative to the effective time values on technical-tactical actions (e.g., passes, dribbles, shots, goals, ball recoveries, and losses, won challenges, and fouls) and internal and external load variables, and the difference between players who are highly and lowly involved during match play.

The issue about absolute and relative data is great of interest in sport science nowadays (7–10). Relative training and match load may help practitioners to individualize training loads and ensure that players are trained and competed accordingly, considering the relative time that futsal players are on the court. Some studies (7, 9) demonstrated inconsistencies between absolute and relative terms in sports. For example, Douchet et al. (7) evaluated absolute and relative training load, (i.e., relative load calculated by dividing absolute training load by mean values from the competitive matches) and compared across playing positions during a full in-season in an elite soccer academy, found that training load for central defenders was underestimated using absolute training loads for moderate and high-speed distances, however, relative training loads highlighted wide midfielder as an underloaded position. Given that, the same author (7) concluded that relative training load is recommended as it is contextualize training load according to competitive demands and favor training individualization. However, when it comes to futsal, no previous studies have been researched about the physical and technical-tactical demands considering absolute and relative values.

To the best of the authors' knowledge, this study represents the first attempt to interpret match performance in elite futsal players with different involvement (i.e., actual participation in match play considering playing time, internal and external load variables), and normalize the absolute values per effective time. The main aim was to analyze the differences in absolute and relative individual tactical actions, internal and external load variables between players with high- (HIGH) and low-involvement (LOW) (i.e., higher or lower effective playing time, session rate of perceived exertion [sRPE], and player load [PL]) during match play. It was hypothesized that HIGH players would present higher absolute values on physical and tactical-technical actions when compared to their LOW counterparts because of the higher time spent in the court, but non-significant or opposite from the absolute values differences would be found between groups considering playing time (i.e., relative values) as it has been found in other studies (7, 9).

## 2. Methods and materials

### 2.1. Study design

A retrospective observational study was designed. The study followed a rigorous design, normalizing the variables using effective time, to explore the differences between time variables (absolute vs. relative values) on various performance indicators, such as PL, sRPE, fouls, shots, shots on target, goals, successful dribbles, passes, successful passes, ball losses, ball recoveries, and challenges won in the context of competitive futsal match play.

### 2.2. Participants

A total of 15 official matches involving the Portugal National Team in three distinct competitions were included in this study. The data were collected from the following events: FIFA Futsal World Cup 2021 = 7 matches; UEFA Futsal Euro 2022 = 6 matches; Futsal Finalíssima 2022 = 2 matches. A statistical package (Jamovi, version 1.8, 2021) was used for the calculation of the sample size. One sample T-Test was used to calculate the N. A minimally-interesting effect size (ES) (δ) 0.85, probability of error of 0.05, and minimum desired power 0.8, a sample size of 13 futsal players were required, similar to previous futsal studies (3, 11, 12). Thirteen different players in total (age: 27.4 ± 4.7 years, weight:70.3 ± 7.6 kg, height:176.3 ± 5.5 cm) participated in this investigation (10 same players participated in the FIFA Futsal World Cup 2021 and UEFA Futsal Euro 2022 and 3 new players called for the Futsal Finalíssima 2022 matches). Inclusion criteria were the following: (1) outfield player, and (2) no report of physical limitation or skeletal muscle injury that could affect performance, and (3) only data from participants who played ≥ 2 min were analyzed, as previous studies applied (3, 11). Goalkeepers were not included in this study due to its performance specificities. Ethical considerations were addressed, and the study adhered to the guidelines and regulations for human research ethics of the Declaration of Helsinki and was approved by the local Ethics and Scientific Committee with code CE-UBI-Pj-2020-043:ID162. Data anonymity and confidentiality were maintained throughout the study, ensuring the privacy and rights of consenting athletes.

### 2.3. Procedures

Physical performance variables were assessed using indicators of external and internal load. The external load variables were collected in all matches using wearable inertial measurement units with ultra-wideband tracking systems technology (WIMU PROTM, Realtrack Systems SL) at a sampling frequency of 100 Hz. The units were turned on about 30 min before the warm-up and worn by players in a specific custom neoprene vest located on the middle line between the scapulae at C7 level. Due to tournament regulations, only PL (13) was consistently recorded as an external load variable during the competitions, as the use of antennas was not allowed. PL is the vector sum of device accelerations in its 3 axes (vertical, anteroposterior, and lateral). Data analysis was performed using corporative software (SPRO, Realtrack Systems SL). The devices' accuracy and reliability have been previously validated and reported (14).

For the assessment of internal load, RPE and sRPE was registered 30 min after the end of each match using the adapted scale of Foster (15). Players received a short questionnaire on their smartphones using Microsoft Forms (forms.office.com) with a 10-point visual analogue scale to rate the perceived intensity of the match, ranging from 0 (“not at all”) to 10 (“maximum effort”). Prior to data collection, all players were familiarized with the scale. The sRPE was calculated by multiplying the RPE score by each player's total effective playing time. The use of RPE as a measure of internal load in futsal has been successfully implemented in previous studies (12, 16, 17).

Individual tactical action variables were extracted from match-statistics, selected, and organized into a unified matrix using an Excel spreadsheet. InStat Scout® is a commonly used computer tool for data analysis in futsal competitions and is often employed by scouting services in elite futsal leagues. This tool has been previously utilized in futsal literature (18) to analyse player performance and game dynamics. The variables for measuring individual tactical actions included fouls, shots, shots on target, goals, successful dribbles, passes, successful passes, ball losses, ball recoveries, and challenges won. As a widely adopted video analysis tool in sports, InStat Scout® provides objective data and performance variables, and has been previously used in futsal literature to analyse player performance and game dynamics (18).

All the data were measure using two different approaches:

(i) absolute: this approach encompassed all periods, even instances when the clock was paused. It included the total PL and the number of technical-tactical actions per player throughout the entire duration of the match.
(ii) relative: this approach involved normalization based on effective time, which accounts solely for periods when the ball was in play and the clock was active. It encompassed the PL and the number of actions per minute of playing time.

### 2.4. Statistical analysis

A statistical package (Jamovi, version 1.8, 2021) was used for the statistical analysis. A descriptive statistic with mean ± standard deviation was performed for all the variables. A total of 149 data points, separated into 74 data points for group HIGH and 75 for a group LOW for the analysis. Athletes were classified based on their performance profiles using a two-step cluster analysis, employing log-likelihood as the distance measure and Schwartz`s Bayesian criterion (19). The cluster analysis classified the players into two distinct groups, namely HIGH and LOW, based on their average effective playing time (volume), sRPE (internal load), and PL (external load). A linear mixed model was used to examine the differences in external and internal load and tactical variables between HIGH and LOW, accounting for individual repeated measures. Cohen's d ES and 95% confidence intervals (CI) were performed and interpreted as follows: <0.2 trivial; 0.20–0.59 small; 0.60–1.19 moderate; 1.2–1.99 large; and ≥2.0 very large (20). A significance level was set at *p* < 0.05.

## 3. Results

The HIGH cluster, representing 49.7% of the sample, displayed a strong association with the predictor variables, with effective playing time, sRPE, and PL having predictor's importance of 1.00, 0.99, and 0.73, respectively. Similarly, the LOW cluster comprised 50.3% of the sample and exhibited the same predictor`s importance values as the high-involved cluster. The average silhouette measure of cohesion and separation, indicating the quality of the clustering, was 0.6, indicating a good quality of the model.

Table 1 compares the absolute variables between the groups (HIGH vs. LOW). Considering absolute variables, HIGH players presented higher sRPE, RPE, total PL, shots, goals, passes, successful passes, ball losses and recoveries, and lastly, challenges won (*p* = 0.001–0.039; ES = 0.43–2.48) than LOW players. Conversely, no differences were observed in fouls, shot on target, and successful dribbles (*p* = 0.72–0.169; ES = 0.35–0.46).

**Table 1:** Comparison of the absolute variables between the groups.

Variables	Units	Groups		*p*-value	ES (95% CI)
		HIGH	LOW		
sRPE	a.u	159 ± 47	65 ± 26	0.001*	2.48 (1.51–2.41)
RPE	a.u	8.2 ± 1.5	6.5 ± 1.6	0.001*	1.08 (0.73–1.42)
Player load	a.u	94 ± 18	62 ± 13	0.001*	1.97 (1.97–2.99)
Fouls	n	0.8 ± 0.9	0.5 ± 0.8	0.169	0.35 (0.02–0.67)
Shots	n	4.2 ± 2.7	2.9 ± 2.6	0.028*	0.51 (0.17–0.84)
Shot on target	n	1.7 ± 1.4	1.0 ± 1.2	0.072	0.46 (0.13–0.79)
Goals	n	0.3 ± 0.5	0.1 ± 0.4	0.039*	0.43 (0.10–0.75)
Successful dribbles	n	0.9 ± 1.1	0.5 ± 0.7	0.083	0.43 (0.10–0.76)
Passes	n	50 ± 24	28 ± 14	0.001*	1.11 (0.74–1.48)
Successful passes	n	43 ± 21	24 ± 13	0.001*	1.07 (0.70–1.43)
Ball loses	n	4.9 ± 2.6	2.3 ± 1.8	0.001*	1.16 (0.79–1.53)
Ball recoveries	n	3.4 ± 2.6	1.9 ± 1.7	0.001*	0.70 (0.35–1.04)
Challenges won	n	5.1 ± 1.8	2.7 ± 1.8	0.001*	1.01 (0.64–1.36)

Data presented as mean ± standard deviation. Each data point represents the average value per player.

a.u, arbitrary unit; HIGH, high-involvement; LOW, low-involvement; sRPE, session perceived exertion.

* Significant difference (*p*-value <0.05) between the two groups.

Table 2 compares the relative variables normalized per effective time between the groups (HIGH vs. LOW). Interestingly, when analyzing data relative to effective playing time, LOW players reached higher total PL and shots per minute (*p* = 0.001–0.012; ES = 0.39–0.93) when compared to HIGH players. Non-significant differences were found for the rest of the variables.

**Table 2:** Comparison of the relative variables per minute between the groups.

Variables	Units	Groups		*p*-value	ES (95% CI)
		HIGH	LOW		
Player load·min^−1^	a.u	4.95 ± 1.16	6.93 ± 2.75	0.001*	0.93 (0.57–1.28)
Fouls·min^−1^	a.u	0.04 ± 0.05	0.05 ± 0.08	0.151	0.13 (−0.18–0.45)
Shots·min^−1^	n	0.21 ± 0.12	0.29 ± 0.23	0.012*	0.39 (0.07–0.72)
Shots on target·min^−1^	n	0.08 ± 0.07	0.10 ± 0.11	0.195	0.20 (−0.12–0.52)
Goals·min^−1^	n	0.02 ± 0.03	0.01 ± 0.04	0.695	0.06 (−0.38–0.25)
Successful dribbles·min^−1^	n	0.05 ± 0.06	0.04 ± 0.07	0.992	0.06 (−0.38–0.25)
Passes·min^−1^	n	2.47 ± 0.92	2.81 ± 1.05	0.052	0.34 (0.01–0.66)
Successful passes·min^−1^	n	2.14 ± 0.84	2.44 ± 0.97	0.100	0.32 (0.00–0.65)
Ball losses·min^−1^	n	0.25 ± 0.12	0.24 ± 0.20	0.861	0.03 (−0.35–0.28)
Ball recoveries·min^−1^	n	0.17 ± 0.12	0.20 ± 0.19	0.324	0.14 (−0.17–0.47)
Challenges won·min^−1^	n	0.27 ± 0.15	0.30 ± 0.21	0.510	0.16 (−0.15–0.48)

Data presented as mean ± standard deviation. Each data point represents the average value per player.

A linear mixed model was used to analyze the differences between the two groups.

a.u, arbitrary unit; HIGH, high-involvement; LOW, low-involvement.

* Significant difference (*p*-value < 0.05) between the two groups.

## 4. Discussion

This study analyzed the differences in absolute and relative internal and external load variables and tactical performance variables between highly- and lowly-involved players during match play. The main findings revealed that when considering absolute values, HIGH players presented higher values in sRPE, total PL, shots, goals, passes, successful passes, ball losses and recoveries, as well as challenges won. However, when variables were normalized using effective time, LOW players achieved higher total PL and shots per minute compared to their HIGH counterparts.

Previous researchers (1, 2, 21, 22) investigated match play demands but only considered the average values of all players participating in competition, without separating those who were more involved (i.e., more minutes, higher PL, and sRPE) from those who were less, as in the present study. Herein, when considering absolute values, HIGH players presented greater PL (94 vs. 62), sRPE (159 vs. 65), and RPE (8.2 vs. 6.5) than LOW players. However, when normalizing per effective time, LOW players presented higher sRPE and PL per minute when compared to HIGH players. This could be explained by the “pacing” strategies that HIGH players may utilize in order to endure across the match, while LOW players (aware of their role) perform at maximal exertion during their (limited) effective playing time (23–25). In practical settings, when sports practitioners analyze external and internal load in futsal, it is crucial to analyze the data considering the interruption of the match-clock whenever the ball goes out of play. The differentiation between total and effective time can have a significant impact on the physical performance of players (11). Therefore, it is advisable to approach the interpretation of both external and internal load data with caution, as the match itself has its own pacing that needs to be recognized, including the temporal relationship between active ball play and periods when the ball is out of play.

Futsal players perform technical-tactical actions with (i.e., passes, dribbles, shots) or without the ball (i.e., tackles, ball interceptions and recoveries) to sustain the collective behavior of the team. Interestingly, this is the first study to report technical-tactical activities of futsal players during the game, normalizing the variables using effective time and separating the players with higher or lower involvement during match play. Considering absolute values, a HIGH futsal player performs significantly more shots (4.2 vs. 2.9), goals (0.3 vs. 0.1), passes (50 vs. 28), successful passes (43 vs. 24), ball losses (4.9 vs. 2.3), ball recoveries (3.4 vs. 1.9), and challenges won (5.1 vs. 2.7) per game. However, when the current variables were normalized per effective time, LOW players performed more shots per minutes when compared to HIGH players. From an applied perspective, sports practitioners in futsal are advised to evaluate a player's tactical-technical performance considering absolute but mainly relative values, as it is “logical” and expected to have higher numbers of total shots, passes, etc., with more playing minutes and the relative approach may allow drawing more meaningful analyses.

This study has limitations in terms of sample size, as only one futsal National Team was analyzed. A second limitation is related to the players' high level of expertise (i.e., champions of prestigious international tournaments) that may restrict generalizability. Thus, the results could be different in other futsal teams, with other players and competitions. Additionally, the monitoring of external and internal load was limited to PL and sRPE. Other physical and technical-tactical variables, such as high-speed running, accelerations, decelerations, heart rate, playing position and tactical role could provide further insights on the influence of absolute values and when considering effective playing time.

In summary, these findings emphasize the importance of normalizing match performance data using effective playing time in futsal, as players with lower involvement may present similar or even superior physical and technical-tactical performance compared to teammates with higher playing time. During data visualization and analysis, coaches should be aware about the data normalization in order to not sustain their decisions based on biased analysis. Instead of looking to the number of efforts or actions, coaches should base their decisions of performance based on the performance of players per minute. Also, further research should be developed in order to understand the variation of players' performance between rotations.

## Data Availability

The raw data supporting the conclusions of this article will be made available by the authors, without undue reservation.
